# Dementia and patient safety in the community: a qualitative study of family carers’ protective practices and implications for services

**DOI:** 10.1186/s12913-019-4478-2

**Published:** 2019-09-05

**Authors:** Kristin Häikiö, Mette Sagbakken, Jorun Rugkåsa

**Affiliations:** 1 HØKH – Health Services Research Unit, Akershus University Hospital, Postbox 1000, 1478 Lørenskog, Norway; 2 Oslo Metropolitan University, Pilestredet 32, 0166 Oslo, Norway; 3 Centre for Care Research, University of South-Eastern Norway, 3900 Porsgrunn, Norway

**Keywords:** Dementia, Alzheimer disease, Family caregiver, Patient safety, Primary health care, Health care quality, access and evaluation, Frail elderly, Community health services

## Abstract

**Background:**

Dementia is a cause of disability and dependency associated with high demands for health services and expected to have a significant impact on resources. Care policies worldwide increasingly rely on family caregivers to contribute to service delivery for older people, and the general direction of health care policy internationally is to provide care in the community, meaning most people will receive services there. Patient safety in primary care is therefore important for future care, but not yet investigated sufficiently when services are carried out in patients’ homes. In particular, we know little about how family carers experience patient safety of older people with dementia in the community.

**Methods:**

This was an explorative study, with qualitative in-depth interviews of 23 family carers of older people with suspected or diagnosed dementia. Family carers participated after receiving information primarily through health professionals working in dementia care. A semi-structured topic guide was used in a flexible way to capture participants’ experiences. A four-step inductive analysis of the transcripts was informed by hermeneutic-phenomenological analysis.

**Results:**

The ways our participants sought to address risk and safety issues can be understood to constitute *protective practices* that aimed to prevent or reduce the risk of harm and/or alleviate damage from harm that occurs. The protective practices relate to four areas: physical harm, economic harm, emotional harm, and relational harm. The protective practices are interlinked, and family carers sometimes prioritize one over another, and as they form part of family practice, they are not always visible to service providers. As a result, the practices may complicate interactions with health professionals and even inadvertently conceal symptoms or care needs.

**Conclusions:**

When family caregivers prevent harm and meet needs, some needs may be concealed or invisible to health professionals. To recognize all needs and provide effective, safe and person-centered care, health professionals need to recognize these preventive practices and seek to build a solid partnership with family carers.

## Background

Dementia is a chronic and progressive disease that leads to deterioration in memory, thinking, behavior and the ability to take part in daily activities [1]. It is strongly associated with old age [1–3], and symptoms in the early stages often go undetected [4]. Dementia is a cause of disability and dependency [5, 6] and associated with high demands for health services, with significant impact on resources [7–12]. Despite fewer risk factors in later-born people and a reduction of age-related dementia, the prevalence of dementia is expected to increase in Norway [13] and elsewhere [5, 14], due to the growing number of older people in the population [15–17]. The general direction of international health care policy is increasingly to provide care in the community, which, for those affected by dementia in Norway, often means in primary care, nursing homes and various services provided in people’s own homes. Simultaneously, care policies increasingly rely on family caregivers to contribute to service delivery to older people. Policy documents point out that new ways of organizing health service delivery in cooperation with family carers must be found to meet future demands of dementia care in a sustainable way for all parties [18–21]. Guidelines for family carer involvement have been developed in Norway [22] and elsewhere [23, 24], and many national dementia strategies highlight the importance of supporting family carers to maintain their capacity to engage in such roles over time [25, 26]. In order to understand how these policies are implemented on the ground, it is important to understand family caregivers’ perspectives and expectations in their own rights, even though these might differ from those of care-recipients.

Patient safety has been another key area of health policy worldwide since the late 1990s [27, 28]. Discussions about patient safety have moved beyond a concern for avoidable medical errors and hospital mortality to now also direct focus on broader issues of how to maintain quality of life and dignity in health service delivery, both in hospital and community settings [27]. Related to community-based dementia care, identified safety issues include falls, food safety, traffic safety, wandering around disorientated, and polypharmacy [29, 30]. Despite the shift towards care in the community, little is known about how patient safety is practiced in this setting [31–33]. Importantly, there is a dearth of research in the perspective of family carers of what constitutes risk of harm to persons with dementia and how family carers seek to address these risks [31, 32, 34]. Given their increased role in health service delivery, this lack of knowledge is a concern, not least because family carers, patients, and health professionals may have different perspectives on these issues. In this article, we examine family carers’ perspectives on how to prevent different forms of harm to those living with dementia while receiving community-based services, and how their efforts to alleviate those risks might affect and interact with health professionals’ activities in this regard.

## Method

### Setting

Norway has a publicly funded health service available to all citizens [35]. The more than 400 municipalities are responsible for primary care, which typically includes home care, nursing homes and general practitioners. Specialist care is provided by regional health authorities and consists of hospitals and specialized units, such as memory clinics and geriatric outpatient clinics [36]. The principle of lowest effective level of care was introduced in Norway in 1974 [37, 38]. Therefore, older people living with dementia receive services while living at home when possible.

### Design

Given the lack of previous research on family carers’ experience and contributions to preventing harm to older people with dementia, and their interaction with health services, we designed an exploratory study. Semi-structured qualitative interviews were used to maintain a flexible and open approach that would allow participants to talk about issues relevant to them. We developed a topic guide, based on existing research, with themes such as carers’ contributions and interactions with health services, service integration and quality of services, and burdens or benefits they experience. This ensured we discussed the same topics with all interviewees. The guide was applied in a flexible manner, allowing the interviewer (KH) to follow up on clues and turns in the interviews, which helped us capture nuances and reflections expressed in participants’ own words and framed in the context of their lives [39].

We consulted a user panel of 11 people with personal and/or professional experience from a range of health services, institutions or organizations, on study design, recruitment strategy and developing the topic guide. A pilot interview was conducted with one panel member, and the topic guide and interview approach were found to work well.

### Definitions and descriptions of the sample

The main inclusion criteria were that interviewees should be an informal carer for a family member, neighbor or friend aged 65 years or older who received health services due to symptoms of dementia. Dementia was defined as symptoms of dementia with or without a formal diagnosis. If diagnosed, all forms of dementia were included. We wanted to obtain maximum variation [40, 41] in the experiences of caregiving and therefore sought men and women with different relationships to the person with dementia, living in rural and urban areas, and born in Norway and abroad.

We sampled in three phases. In the first phase, health professionals who worked with older people with dementia passed along information about the study to potential participants on our behalf. From this, we obtained contact details of potential participants, and others contacted us directly. We also spread information through the study’s Facebook page. We interviewed the first ten participants who volunteered through these methods, the majority of whom were women and spouses of persons with suspected or diagnosed dementia. To balance the sample, in the second phase, we asked health professionals specifically to invite male participants, non-spouses and people born outside of Norway. We also engaged in targeted snowballing through earlier participants and personal networks and recruited five additional participants. After these first 15 interviews, few new experiences or information emerged. However, because all participants lived in the eastern part of Norway, in the third phase, we recruited six additional participants from the northern part of the country. This was again through health professionals and snowballing. This added the perspectives of caregivers with Sami (indigenous) background and participants from rural, small communities with vast distances to specialist services. Of the 26 people recruited, three cancelled before the interviews were conducted, two due to acute illness, and one did not give a reason. In the final sample of 23 participants, some cared for people with symptoms of advanced dementia, while others were in the phase where they suspected dementia or of ongoing medical investigations to reach a diagnosis. The majority of our sample are female, spouses and middle aged. Overall, an acceptable degree of breadth in participants’ characteristics was achieved, as shown in Table 1.

**Table 1:** Characteristics of the sample at the time of the interview, *n* = 23

Characteristics	*n* = 23	
Gender, n (%):		
Female:	17 (74)	
Male:	6 (26)	
Age, years min-max (median):	44–83 (62)	
Relationship, n (%):		
Spouses	12 (52)	
Adult children	9 (39)	
Adult siblings	2 (9)	
Geography^a^, n (%):	Urban areas, *n* = 14 (61)	Rural areas, *n* = 9 (39)
North of Norway, *n* = 6	0	6
East of Norway, *n* = 17	14	3
Living arrangements, n (%):		
Shared household with the person with dementia		11 (48)
Not sharing household with the person with dementia		6 (26)
Care recipient lived in nursing home		6 (26)

^a^Rural areas = municipalities with less than 20.000 inhabitants, Urban areas = municipalities with more than 20.000 inhabitants. We classified patients’ home municipality into rural and urban based on a combination of population density and proximity to regional centers and other towns/cities first calculated by Rugkåsa et al. [42] and available on request

Collectively, the participants had experience with a variety of services, including nursing homes, home care nurses, day care centers, walking buddy services, volunteer visitors, food delivery services, personal assistants, home help, dementia teams, general physicians, pharmacists, psychologists, physiotherapists, as well as non-government organizations and interest-groups.

### Data collection and analysis

Data was collected between June and October 2017. The first author was the interviewer and a PhD student who had some former experience and training with in-depth research interviewing and qualitative analyses.

Participants chose the time and place of the interviews, which were conducted in participants’ homes, workplace, or in a neutral meeting room or cafeteria with only the participant and the researcher present. It had the character of an informal conversation where the participant was encouraged to speak freely, and KH followed up on clues and new topics relevant to the aim of the study. The interviewer presented herself as a researcher with a nursing background. All participants were informed that what they said would be treated in strict confidence and that they could refrain from questions or withdraw from the interview at any stage. No participants chose to avail of either option. On the contrary, they seemed eager to tell their stories and motivated to contribute to the study’s aims, which gave us rich descriptions of their perspectives. Most interviews lasted approximately 1.5 h. All interviews were audio recorded and transcribed verbatim by the first author. Participants were interviewed once, but one interview was disrupted and continued a week later.

Analyses of the transcripts were informed by hermeneutic-phenomenological approaches [43, 44] and conducted in four analytic stages as shown in Table 2. These were conducted by KH, and discussed in detail with JR. Findings and interpretations were examined and discussed among the authors and feedback was provided by a wider research group, but not by participants.

**Table 2:** Analysis made in four stages, combining different techniques

Stage 1	Stage 2	Stage 3	Stage 4
Transcribing and first impressions	Interim analysis	Inductive coding	Connecting codes and themes
• Interviews transcribed verbatim, usually before the next interview was conducted [48]. • Transcribing while listening shaped initial overall impressions and informed subsequent interviews [47, 48]. • Naïve reading gave an overview of within-case experiences and perspectives [46, 49].	• Memo-writing and the constant comparison method were used to track and elaborate differences and similarities between cases [45]. • Initial interpretative analysis conducted to understand different aspects: ^1)^ describing how participants understood themselves, ^2)^ interpreting the meaning of their narratives, ^3)^ interpreting underlying and hidden interests, hidden agendas and using critical interpretation [44]. • Emerging themes were compared to earlier research.	• NVIVO (v. 11) was used to break the text into smaller units [48]. • Inductive, line-by-line coding resulted in 1383 descriptive and interpretive codes [45, 46]. • These were organized hierarchically in 53 main codes and numerous sub-codes [43].	• Codes were interpreted and abstracted into themes [46, 47] • Mind-mapping in NVIVO connects codes to themes. • Themes that integrated impressions from earlier phases were followed [43]. • A high-level theme of “protecting the person with dementia from harm” was identified. • Codes within that theme were categorized into 4 protective practices described by participants, related to potential physical, economic, emotional and relational harm.

In the presentation below, quotes from the transcripts are included to illustrate and validate our interpretations [50] and, unless otherwise specified, represent common views in the sample.

## Results

From the interviews, we found that some of the ways in which our participants supported the person with dementia can be understood to constitute *protective practices* aimed to prevent or reduce the risk of harm and or alleviate damage from harm. The protective practices relate to four areas: physical harm, economic harm, emotional harm, and relational harm. We describe each and then further elucidate how these protective practices are interlinked, how family carers sometimes prioritize one over another, and how these practices may complicate interactions with health professionals and even inadvertently conceal symptoms or care needs.

### Preventing physical harm

The first area of protective practices involved various ways family carers sought to protect the person with dementia against potential physical harm. Sub-themes include 1) preventive presence, 2) tailored use of protective aids and 3) monitoring of health professionals.

#### Preventive presence

Visiting or being present with the person with dementia, continuously or frequently, was considered an important preventive measure because it enabled participants to react immediately, prevent harm such as falls or accidents at home or in traffic, and limited the consequences of physical harm that did occur. Those living in the same household as the person with dementia were often present with the person in daily tasks such as helping out with grooming, organizing meals, eating together, assisting or guiding the person when walking around in the house or outside. Hannah explained her own role in preventing physical harm to her husband by being present.

*If I’m going anywhere, I always have someone look after him. … So that’s like, I feel I am on guard the whole time. That I am, well, waiting for some noise. Being alert all the time.* (Hannah, 62, caring for her husband)

Others too said that they could not leave the person with dementia alone and asked someone to come when they needed to leave the house. Those who lived separately usually paid frequent visits or phoned to confirm everything was all right. If the person living with dementia did not answer the phone, the family carer would often stop by. Some explained that the person with dementia would remove their portable alarm (often worn as a watch or a necklace) when going to bed or taking a shower and then forget to put it back on and consequently be unable to call for help. The carer would, therefore, routinely remind the person with dementia to put the alarm back on or make sure that the phone was in good order, with easy access to the most important telephone numbers, such as the number to the family carer.

As symptoms varied from day to day, carers needed to be present and able to adapt to shifting needs and tailor their support. This was, for many, one of the most important safety measures. However, being present meant different things for different carers. For some, it meant being together at the same place and guiding the person with dementia through the day, or monitoring by phone. It could also relate to specific activities, such as being present when the person with dementia was driving. Lenita described how she was worried about her husband’s driving and assisted him in handling it.

*I feel I always need to be his co-pilot and keep an eye on things. … So it’s like, him needing to focus on the driving and that, and I need to, like, navigate.* (Lenita, 61, caring for her husband)

Several participants who lived separately from the person with dementia, worried about the time lapse between visits from home care nurses and compensated with frequent visits and phone calls. This indicates that family carers sometimes considered health services to be insufficient in protection against physical harm. Participants sometimes wanted to involve health professionals in protecting against such risk. This was sometimes difficult because the person with dementia gave a better impression to the professionals than they did in their daily life at home. For example, the persons living with dementia could “pull themselves together” during meetings, and symptoms could vary during the day or between “good and bad days”. Elinor said her father’s GP referred him for a renewal driving test. On that day, her father pulled himself together and passed the test, which Elinor found distressing.

*He had to go out with a driving instructor. And on that day, he had a good day. He’d slept well, got his coffee, and he drove like a prince. But it was only a week before he almost crashed into an ambulance (laughs) because he didn’t heed the right-of-way.* (Ellinor, 49, caring for her father)

Other participants also said that since they were the ones knowing the person with dementia the best, they detected needs that were not easily discovered by others, and it could be difficult to communicate the scope of the situation to professionals who were not present as much.

#### Tailored use of protective aids

Health services frequently provided a range of aids to protect against physical harm, such as stove guards, electronic calendars, portable alarms, or single-dose medication containers. While these can be helpful, many participants expressed that such protective aids often were offered too early or too late in the illness trajectory, or that they were not always tailored to the situation of the person living with dementia and their household. Grethe explained how gadgets they would not use were distributed routinely to her husband. She put them at the back of the cupboard and found her own techniques to manage.

*Grethe: And they came with this calendar … You plug it in, and it shows you the day and date. I’ve pushed that to the back of the cupboard … We don’t need it. Because I put a note on the fridge that is a reminder to both him and me about what day it is. And that works for both of us *… *And then they gave us a stove guard *… . *That probably works really well for those who are demented and use the stove *… *Well, it was the local authority who said that you need to have one of those.* *Okay, we said*.

*KH: So, you were not asked about your needs?*

*Grethe: No, we got it delivered.* (Grethe, 79, caring for her husband)

In other cases, illness progression meant gadgets were no longer useful. Jenny’s mother could no longer use the portable alarm provided by services, and instead she called Jenny when she needed help.

*Before, she understood intuitively that she should push* [the button on the personal alarm], *but now she sometimes calls me when she needs help, because she doesn’t understand that she should push it.* (Jenny, 55, caring for her mother)

Finding the right equipment for the right protection at the right time was challenging, and family carers often found their own solutions. Eva, living with her husband with dementia disease in the north of Norway, implemented a creative solution to prevent him from leaving the house unattended and risking hypothermia.

*Then someone told me, why don’t you hang a bell over the entrance door … so that I wake up. Because I sleep so deeply that I haven’t heard that he’s put his shirt on and gone out … ”.* (Eva, 71, caring for her husband)

For persons living with dementia in their own homes, continuous surveillance such as GPS tracking, camera surveillance or other forms of real-time observations of the person living with dementia were mentioned as potentially useful, but these were not offered from health services.

#### Monitoring health professionals

Some variation in the degree of trust was expressed vis-à-vis health professionals. While some participants said services were helpful in protecting patients against physical harm, others described situations where care was suboptimal or even downright dangerous. Vera talked about her experience with several incidents at the local health service institution.

*When she was at the short-stay unit it was like, “I wonder how she’ll look when I collect her this time. Is she blue and black?*” *Because there were cuts here and there … And once when I collected her … when I took of her pantyhose there was a big cut like this* [indicates 6 cm across the knee]. *So I call the unit and ask if they’ve seen this big cut. You know, it’s not, it’s really big. She needed stitches. “She has had a shower today, but didn’t you notice the cut?” None of them had actually seen it.* (Vera, 49, caring for her mother).

Several family carers explained such unsafe care with staff having too little time and resources, often combined with insufficient, or no, training. Due to such experiences, some said they needed to monitor health professionals. This could be done by visiting at unpredictable hours or routinely asking detailed questions about daily routines to make staff aware that they paid attention. This was the case for Vera when her mother eventually was moved to a nursing home.

*Sometimes I go late, sometimes in the morning, and sometimes in the middle of the day, I just stop by, just to check …. So I feel that I’m a bit of a control freak. Cos I don’t trust them 100% …* (Vera, 49, caring for her mother)

The monitoring of health service personnel, and making their attention known, was described by most carers and added pressure to staff to increase patient safety and care quality.

### Preventing economic harm

Protective practices related to prevention of economic harm surrounded how participants helped the person living with dementia manage their finances. Sub-themes that emerged were 1) practical assistance, 2) monitoring and preventing unnecessary spending and 3) taking full responsibility.

#### Practical assistance

The person living with dementia’s inability to handle internet banking, personal identification number (PIN codes) and other financial transactions was mentioned by many participants and represented a potential threat of economic harm. Many participants assisted in these matters from the early stages of the disease, sometimes before dementia was discovered. For many, like Jenny, her involvement in financial matters gradually increased as the illness progressed.

*She can’t manage internet banking, so I’ve paid her bills for many years. And withdrawn* [money] *and organized food, so I take care of everything.* (Jenny, 55, caring for her mother)

Economic assistance usually developed over time without formalizing access to bank accounts or internet banking. Most spouses found this unproblematic, as they had long histories of shared economic responsibilities. Those who had formalized their access said the process was cumbersome, such as Kjersti when she suddenly took over the care of her father with dementia after her mother died.

*“You see, my dad is demented and can’t handle this … but there is an application in at the moment about him not being able to, or that I will be his guardian and that”. “Yeah,* [continues in a sarcastic tone] *but you’re not allowed to use his online bank codes, you know”. “No, but who will get it done then?” You know, it’s like,* [these institutions were] *not very understanding.* (Kjersti, 46, caring for her father)

While the banks maintained their security procedures to protect their customer against economic harm, the family carer had no immediate way of implementing their practices of protection against economic harm.

#### Monitoring and preventing unnecessary spending

As the dementia progressed, several participants said that they needed to intervene to prevent the person with dementia from spending money unwisely. Ellinor explained that her mother’s ways of handling her money and credit cards left her vulnerable, and Ellinor needed to monitor her spending.

*She hasn’t a clue about her cash point card. She doesn’t know a single PIN code …. She lost her purse, her card …. So she always has to take out that note with her PIN code on, which is in her purse together with her card. We are really scared. So we’ve got to keep an eye on her account, and if there is too much money there, we’ll transfer it, in case she loses it again, you know.* (Ellinor, 49, caring for her mother)

Another reason for monitoring spending was vulnerability to abuse by others, such as telesales persons. There were many examples of how persons with dementia had been persuaded to accept subscriptions on books, magazines or services they did not need, value or understand. Trine had to end several of her sister’s subscriptions purchased this way.

*She’s spent a huge amount of money on rubbish. We’ve discovered she was paying for three TV licenses. … For a while she was getting all these books and she didn’t know she’d said yes to them* [offers made by salespersons by telephone]*. And then we had to fix all of that.* (Trine, 77, caring for her sister)

#### Taking full responsibility

Protective practices in financial matters usually developed within families over the years. None of the participants mentioned that monitoring the handling of money was something they discussed with health professionals in the early stages of the disease. The most common reason to involve health services in an attempt to prevent economic harm was when they chose to apply for legal guardianship. Only a small number of participants had considered getting such guardianships or other formal arrangements in place and most managed well without. Some participants said it felt difficult to deprive a person of the right to handle his or her money. Therefore, it was only after long periods of difficulty that they finally sought advice from professionals about these matters. Daniel (55), caring for his brother, explained that he sought the help of his brother’s GP to obtain legal guardianship after years of assisting and monitoring his brother’s bank accounts. Bettina (70) was among the few participants with several powers of attorney in place even before her husband got ill. She had since extended the range of things she could legally do on his behalf.

*I had formal access to all his accounts, long before he got ill … But in 2010, he became 100% disabled and then he said that we’ve got to set up the power of attorney. So we signed the papers where I am allowed to check … without him present.* (Bettina, 70, caring for her husband)

### Preventing emotional harm

All participants emphasized the importance of preventing emotional harm, and this was done in different ways. Sub-themes were 1) maintaining respect and dignity, 2) preventing loneliness, 3) avoiding negative feelings and 4) promoting good moments and positive feelings.

#### Maintaining respect and dignity

Maintaining dignity and respect was important for how family carers interacted with both the person with dementia and health professionals. For example, to respect the integrity and wishes of the person with dementia, the carer often found it difficult to correct them when they provided inaccurate information to health professionals. Lenita said she tried discretely to include the necessary information about her husband’s diagnosis when she went with him for his first visit with a new GP.

*She* [the GP] *didn’t know that he had Alzheimer’s. But I sort of weave it into the conversation without me opening the door and starting off with “here is a patient with Alzheimer’s”.* (Lenita, 61, caring for her husband)

While most participants valued the services provided by nursing homes or day care centers, some worried that the person with dementia would not be met with respect or consideration for their emotional needs. For several family carers, this was so concerning that they chose to limit the use of some services. Eva (71) said that her main reason for caring for her husband at home was her worry that disrespectful comments and treatment in the nursing home would harm his integrity and dignity. They were both of Sami origin, and she gave an example of behavior she thought would not be respected or understood by non-Sami professionals.

*When he gets to his bed, he does like this* [indicates spitting left and right in accordance with Sami protective traditions]*. But that doesn’t matter, let him live like that … , cos we’ve grown up in the same culture and know our Sami culture … and I think that is tremendously important. … that’s why I don’t want anyone to say this it just nonsense. Cos he lives the way he was taught.* (Eva, 71, caring for her husband)

Among others from minority backgrounds, worries were often related to insufficient knowledge about, or respect for, their traditions and a lack of agreement about what was important and acceptable. Among all participants, concern about emotional wellbeing such as dignity and integrity was usually expressed as a lack of respect for the person “behind the disease”. It was important for the family caregiver that the person living with dementia was treated like a person worthy of the same respect and dignity as everyone else, despite their changed behavior.

#### Preventing loneliness and other negative feelings

Loneliness was a common theme in discussions about emotional wellbeing, and many carers made great efforts to prevent the person they cared for being lonely, such as visiting regularly. Many participants also perceived health services to address loneliness by offering day care centers, activity groups, visiting partner, or drop-in centers. However, the person with dementia often resisted these services, and the family carer tried to get around this resistance. Caroline reflected upon this.

*If we’d keep listening to them* [persons with dementia], *they’d be sitting alone in their house or flat until they rot. I mean, loneliness is worse than the disease maybe. … Don’t let them sit there alone even if they claim they’d rather.* (Caroline, 53, caring for her mother)

Family caregivers also sought to prevent other negative emotions for the person with dementia. Many spoke about how the care recipient’s irrational behavior and resistance against daily activities were hard to deal with. This could cause irritability, and they did their best to prevent these feelings from affecting the person living with dementia. A few carers, such as Hannah, sought advice from, or learned from, health professionals about how they could deal with this issue.

*When* [the dementia team] *comes, I think they are so pleasant towards him. I’m the wife and can get a bit irritated, but I think I learn a lot from them, from how they talk to him.* (Hannah, 62, caring for her husband)

Many carers also learned to deal with negative feelings from other family carers they met in organized support groups or from family carer academies (*pårørendeskoler*). However, in many cases participants also spoke about lack of knowledge among staff, and how to avoid confrontations, conflicts and negative interactions with the person living with dementia by using different techniques, including white lies.

*When I sit here in the common room and see some of* [the health professionals] *working with* [the patients] *say that we’re going to bed now, and when they refuse, rather than phrasing it a bit differently, if they know that the person likes to go shopping for instance, they could rather say that. … you have to keep on playing tricks. Yes, you become skilled at lying (laughs).* (Vera, 49, caring for her mother).

#### Creating good moments and positive feelings through activity

Many carers spoke about how difficult it was for the person with dementia to accept the decline in everyday functioning, and how this could lead to sadness and depression. To combat this, many tried to create positive experiences. Line used to give her husband “good moments” by working in their garden together.

*So, I take out the hedge trimmer and charger and stuff, and he runs around and wants to, thinks he needs to set it up with cables and, he doesn’t really understand that it doesn’t need plugging in (laughs). But then I’ve got it going and he can keep at it. … And then he is very pleased with himself afterwards.* (Line, 79, caring for her husband).

Several carers spoke about positive experiences through activities that could bring them closer together, and prevent boredom, feeling useless, restless and sometimes prevent aggression.

### Preventing relational harm

Many participants spoke about how dementia led to changes that could be harmful to social relationships. Many tried to prevent loss of social relationships and contribute to forging new social relationships for the person living with dementia. Withdrawn behavior or lack of initiative from the person living with dementia was presented as a threat to social relationships. Mari explained how she first discovered this when she encouraged her father to go with them to the grocery store, but he wanted to avoid his declining memory causing embarrassment.

*So, I say to him: why are you not coming along? No, he didn’t really fancy that … “No, because I know the people in the village, but I don’t remember the names anymore. And then they start to ask about my rein herd, where do you keep them, and I haven’t been up there, so I sort of start making stories up”.* (Mari, 56, caring for her father)

For those at more advanced stages of dementia, relationships could be challenged or damaged by strange behavior. Trine’s sister, for example, frequently woke up the neighbors during the night, and Trine thought this was bound to put a strain on those relationships.

*And then she’d knocked on the neighbor’s door at four o’clock in the morning and that makes people very … when things happen in the middle of the night. That happened many times … So the neighbors weren’t left alone, she could just turn up, late at night or early in the morning.* (Trine, 77, caring for her sister).

Changed behavior could make established relationships difficult, and this was particularly difficult before a diagnosis was set which provided an explanation. Eva explained how the early manifestation of her husband’s illness made him rude, agitated and stubborn towards customers in his grocery shop and how she attempted to reduce the resulting relational harm.

*He started complaining to the customers and doing strange things in the shop and becoming very insistent. And making up different explanations. He was painting the shop outside where we worked, and then he spilled some paint on the* [neighbor’s] *car. And there was a heated argument with her … So I had to pay [compensation], but I paid without him knowing it.* (Eva, 71, caring for her husband)

For others, changed behavioral patterns caused concern that others would perceive the person with dementia as something they were not. Daniel expressed worry that his brother’s fondness for playing with children could be misunderstood.

*He loves playing with kids, anywhere. If he sees kids when we walk about, he starts to joke with them and I say, “Take it easy, maybe the parents … ” You have to be a bit careful.* (Daniel, 56, caring for his brother)

Since the person living with dementia often changed behavior and acted in ways that they would not have done previously, the practices to prevent against relational harm were often about persuading or motivating the person to appear in socially acceptable ways.

### Prioritization and the potential for concealment of needs

In some cases, family carers experienced risk in more than one area simultaneously. In the example above where Eva paid for the damage from the paint her husband spilled without him knowing, she tried to protect him emotionally at the same time as she did not want to damage social relationships. In situations with competing interests, there could be a need to prioritize which potential area of harm should be addressed by weighing up potential costs in another area. Family carers and health service personnel did not always intuitively agree on which potential harm to prioritize. While some carers usually accepted the advice of health professionals, others negotiated the prioritization of protective practices. Solveig expressed that she believed it was socially problematic when health professionals positioned her mother’s new commode chair in her living room to avoid physical harm.

(Her) *legs were so swollen that she couldn’t walk … That’s when the home nurse came with a commode chair to mum, which she actually placed in the living room* [sighs and rolls her eyes]*. And mum and them had the house full of visitors. I told the home nurse that we can’t have the commode chair in the living room. That’s not on.* (Solveig, 44, caring for her mother)

Solveig got the professionals to move the commode chair to a different room, and thus decided the resulting risk of physical harm was secondary to the potential for emotional and relational harm of having the toilet chair in the living room area.

As mentioned above, several carers described how persons with dementia could hide or underplay symptoms in their interactions with health professionals. It was then difficult for the carer to reveal such symptoms without risking emotional or relational harm to the person with dementia. As shown in an earlier example, Lenita included information about her husband’s diagnosis in the conversation with the doctor in a way she hoped would protect her husband emotionally but provide enough information to reveal the need for prevention against physical harm. Kjersti explained that health professionals only saw her father a few hours a week in the day care center. Because he was able to pull himself together for short periods, they were given an impression that his overall daily functioning was far better than she experienced at home.

*They thought he was so nice, like, didn’t get why he was there. But that’s because he was very good at pulling himself together when he met others.* (Kjersti, 46, caring for her father)

In addition, Kjersti routinely cleaned up her father’s flat to make it “respectable” before the home care staff arrived. She realized, however, that this protection against relational harm in effect could conceal the extent of his symptoms and needs from health professionals involved in his care. Kjersti had gradually become aware of this, and had therefore started to take photos to give them, while also addressing her father’s immediate needs.

*And particularly because I did so much, it never came to the fore. But then I stopped. I took pictures of how things look down there* [in her father’s flat], *things he was doing, you know, cutting holes in the carpet because there was someone down there he needed to help up. And there were buckets upside down, and there was a bike he was going to fix, the way it looked with all kinds of stuff and the soiled bathroom and toilet and, you know. And it dawned on me … of course when I was down there, I had to clean it all up, otherwise the flat would have been destroyed and my dad would then be living in a pigsty. And it wasn’t, it was beneath his dignity. I mean, that’s not how we are. And I think many would do the same.* (Kjersti, 46, caring for her father)

In retrospect, Kjersti was able to see that her father’s ability to pull himself together, and her protective practices, prevented health professionals from seeing and understanding the scope of the situation, which could limit their ability to offer appropriate medical support, protective aids or sufficient supervision.

## Discussion

By studying family carers’ perspectives on what constitutes risks to people living with dementia and how they seek to prevent, reduce or alleviate harm, we found that they engage in what we call protective practices in four areas related to physical, economic, emotional and relational harm. This means they are involved in many aspects of care recipients’ lives, making many everyday interventions. This is consistent with findings in earlier studies, showing that the majority of family carers to people with dementia are taking measures to prevent risk behaviors [51, 52]. By co-navigating in the car, cancelling duplicate subscriptions, ensuring the care recipient is well dressed and groomed, or making sure the person with dementia is not left alone without the ability to call for help, our respondents addressed many risks and concerns, some of which overlap with health professionals’ remit, others that do not. While these practices may provide essential support to the person with dementia and to services, they might also have unintended consequences or dynamics. Such dynamics might, in part, stem from the different perspectives of those providing formal and informal care and have implications for how health services – and other public services – collaborate with family caregivers. We discuss these three topics in turn.

### The potential for negative feedback loops

The different protective practices are interconnected and sometimes intertwined, making it necessary to resolve conflict between them or decide which area should be prioritized in a particular situation. Family carers and health service personnel sometimes prioritize differently. Our participants’ stories, as well as previous research, indicate that while family carers often prioritize relational, emotional and economic protection, they perceive health professionals to prioritize prevention of physical harm over other needs [53, 54]. When the four protective practices are weighted against each other and prioritized differently among family carers and health service personnel, it may lead to different solutions and considerations.

**Fig. 1:**
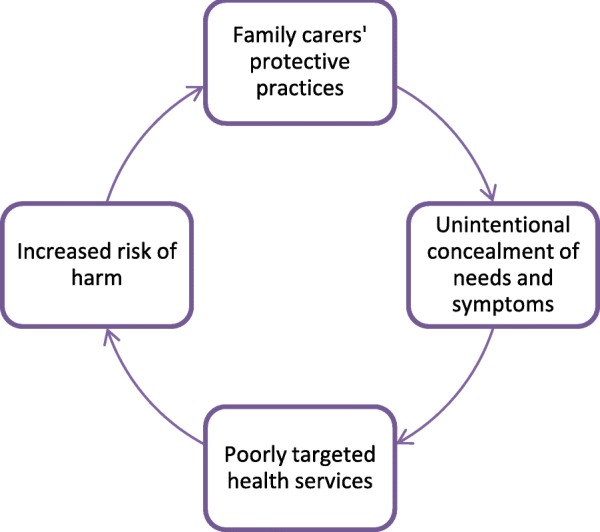
The potential negative feedback loop

Moreover, as carers’ protective practices were usually part of everyday life, they might not be visible to health professionals, who may then not be aware of the full needs of the person with dementia. The effect of the contribution to care by the family carer may also be difficult to see, such as when family carers prevent falls and assist the person with dementia in daily activities. Some protective practices may therefore, in effect, conceal care needs and contribute to gaps between how health professionals and family caregivers perceive the situation. This can, in turn, impact on how targeted the support offered to the person with dementia is. This could, for example, be reflected in the provision of protective aids not suitable to the situation, or in a nursing home not providing food in a way the patient was able to eat. Poorly targeted provision of services can increase the risk of harm to patients by leaving needs unmet, which would trigger family caregivers to continue to engage in protective practices. If, as our participants suggest, they needed to do more as the illness progress, there could be a potential ongoing negative feedback loop where family carers take on increasing responsibility, but their input, and the scope of care needs, remain at least partly invisible to services. Figure 1 depicts such a potential negative feedback loop.

### Difference of perspectives

Participants illustrated how they perceive a wide range of safety risks to persons living with dementia, many of which are not recognized by service personnel. This might be explained by the two very different perspectives from which family carers and health professionals approach their care work. From the perspective of family caregivers, they are involved in supporting, helping and caring for their relative because they are family or friends who share histories, identity and often homes. It is part of family practice [21]. As shown above, our participants seem to approach care in line with WHO’s definition of health as “a state of complete physical, mental and social well-being and not merely the absence of disease or infirmity” [55]. This means their preventive practices for a person with dementia include addressing the individual’s lack of ability to handle one’s private economy or social relationships as well as medication and physical risks.

Health professionals, on the other hand, engage with the person with dementia as part of their professional practice, often over brief periods at the time, during busy shifts. Despite historic roots in a holistic philosophy of care consistent with the WHO’s definition, professional practice today happens within tight boundaries of budgets and time schedules [56–58], and is expected to focus on detecting and meeting specific needs that fall within the scope of their service. Helping with management of personal finance at early stage dementia or maintaining neighborly relationships are usually considered outside the scope of services. Not surprisingly, formal and informal carers may disagree about what constitutes acceptable risks [59] and which safety concern should be given priority in a given situation. However, should a lack of awareness of family carers’ protective practices result in negative feedback loops, it could both produce risky situations that could become services issues over time and impede their ability to provide high quality care that meets patients’ needs. The need for health professionals to be more aware of family carers’ perspectives and develop true partnerships with them is consistent with findings from other studies [53, 60, 61]. Our participants had experience with a range of services, and most service was given with the recipient living at home, often in their homes. This provides health professionals an opportunity to tap into the family practice of which protection against harm forms part. Health care policy is encouraging health professionals to form a partnership with family carers, specifically to provide better-quality care. To be successful in this, family carers’ perspectives, should be considered [25, 26].

### Implication for services

Awareness of how family carers perceive risks, their practices to protect against or alleviate the effects of such risk, and the potential negative feedback loop might be useful to health professionals’ ability to understand the situation of persons with dementia, and in turn, improve the quality of care they provide to this patient group. This seems to require good, ongoing communication between family carers and health professionals and health professionals taking an interest in family practice surrounding patient safety even in areas outside their scope of service. Studies have found that mismatches occur between family carer’s opinions of care needs and public services’ ability to meet these needs through offered services [62–64]. Earlier research suggests that lack of information or awareness of available care and services may be reasons for this mismatch [65], but that the available types of care and service activities’ appropriateness and alignment with needs may also be important reasons [63]. Previous studies have also pointed out that family carers may feel that their knowledge and resources are not utilized by formal carers [66] and that the partnership between them is weak [67]. This study adds to this knowledge by suggesting that better targeted services, tailored through improved partnerships between health professionals and family caregivers and awareness about the concealment of needs, most likely have the ability to be more effective and efficient and can ease the burden on family carers, thus, reducing patient risk. Findings in our and earlier studies [32, 33] suggest that family carers’ protective practices prevent harm in areas within and outside the scope of services. To utilize family carers’ resources, health services need to be aware that family carers’ contribution extends beyond what is covered by, or visible to, health services. When health professionals evaluate family carers’ resources, they must consider their total care contribution. A true partnership with the family carers is needed to be able to see behind the obvious, and tailor services to actual needs. Health professionals need to consider family carers’ wishes to participate and be supportive of the family carer and acknowledge their contributions. Health services can benefit from a partnership which enables an informal carer to continue preventing harm in areas that fall between or outside existing services, while health services can co-exist when demands exceed family caregivers’ capacity [48]. If we are to develop better and safer care for older people with dementia, more research is needed in all aspects of patient safety in primary care [68] as well as in how to build stronger partnerships between family carers and health professionals. It is also important to keep in mind that the views and experiences of the person with dementia may differ from those of their family carer [69].

### Strengths and limitations

A strength of this study is the breadth of our sample as regards gender, age, care roles and geographic context. We deliberately applied a wide definition of dementia, including those with suspected dementia that was not yet diagnosed so as to include the perspectives of family carers at all stages of dementia. The interviews provided rich information about participants’ experiences. It is possible that other methods could have given additional insights. This study does not include the perspectives of people with dementia, which might differ from the perspectives of family caregivers. The interviews were conducted by the main author after having received training in advanced qualitative methods, in close collaboration with the third author. Because our preunderstandings may potentially influence analytic choices and interpretations, these were examined in detail among the authors to reduce potential effect.

## Conclusion

Family carers are involved in various protective practices surrounding physical, economical, emotional, and social harm. These practices illuminate what family carers identify as risks to persons with dementia and what they do to address those risks. As these practices are part of family practice, what they signify is not immediately available to health professionals. Certain practices might inadvertently conceal symptoms and care needs, which in turn could have an impact on how well services are targeted, potentially increasing patient risks. Improved communication and stronger partnerships between family carers and health professionals are needed to prevent such potential negative feedback loops and to improve health care quality for persons with dementia.

## Data Availability

The datasets generated and/or analyzed during the current study are not publicly available due to the privacy of participants and risk of indirect identification by characteristics given in the interviews. Transcripts are available from the corresponding author on reasonable request.

## Abbreviations

KH: The first author, Kristin Häikiö
